# Low but not undetectable early postoperative nadir serum cortisol predicts sustained remission in Cushing’s disease

**DOI:** 10.1530/EO-21-0026

**Published:** 2022-04-07

**Authors:** Anna Stroud, Pearl Dhaliwal, Richard J Harvey, Raquel Alvarado, Benjamin P Jonker, Mark J Winder, Jessica W Grayson, Ann McCormack

**Affiliations:** 1 Rhinology and Skull Base Research Group, St Vincent’s Centre for Applied Medical Research, Sydney, Australia; 2 St Vincent’s Hospital Clinical School, Faculty of Medicine, UNSW Sydney, Sydney, Australia; 3 Garvan Institute of Medical Research, Sydney, Australia; 4 Faculty of Medicine and Health Sciences, Macquarie University, Sydney, Australia; 5 Faculty of Medicine, Notre Dame University, Sydney, Australia; 6 Institute of Academic Surgery, Royal Prince Alfred Hospital, Sydney, Australia; 7 Department of Otolaryngology Head and Neck Surgery, University of Alabama Birmingham, Birmingham, Alabama, USA; 8 Department of Endocrinology, St Vincent’s Hospital, Sydney, Australia

**Keywords:** transsphenoidal surgery, Cushing’s disease, ACTH-secreting pituitary adenoma, pituitary neoplasms

## Abstract

**Objective:**

Transsphenoidal surgery (TSS) is the first-line treatment for Cushing’s disease. The objectives of the study were to determine remission and recurrence rates after TSS for Cushing’s disease, identify factors that predict these outcomes, and define the threshold for postoperative morning serum cortisol (MSeC) that most accurately predicts sustained remission.

**Methods:**

Records were retrospectively reviewed for consecutive adults undergoing TSS for Cushing’s disease at a tertiary centre (1990–2019). Remission was defined as MSeC <138 nmol/L by 6 weeks postoperatively. Recurrence was defined as elevated 24-h urine free cortisol, lack of suppression after dexamethasone or elevated midnight salivary cortisol.

**Results:**

In this study, 42 patients (age 47 ± 13 years, 83% female) were assessed with 55 ± 56 months of follow-up. Remission occurred after 77% of primary (*n* = 30) and 42% of revision operations (*n* = 12). After primary surgery, remission was associated with lower MSeC nadir (26 ± 36 nmol/L vs 347 ± 220 nmol/L, *P*  < 0.01) and lower adrenocorticotropin nadir (2 ± 3 pmol/L vs 6 ± 3 pmol/L, *P* = 0.01). Sustained remission 5 years after surgery was predicted by MSeC <92 nmol/L within 2 weeks postoperatively (sensitivity 100% and specificity 100%). After revision surgery, remission was predicted by lower MSeC nadir (70 ± 45 nmol/L vs 408 ± 305 nmol/L, *P* = 0.03), smaller tumour diameter (3 ± 2 mm vs 15 ± 13 mm, *P* = 0.05) and absence of cavernous sinus invasion (0% vs 71%, *P* = 0.03). Recurrence after primary and revision surgery occurred in 17% and 20% of patients respectively.

**Conclusions:**

Lower postoperative MSeC nadir strongly predicted remission after both primary and revision surgery. Following primary surgery, an MSeC <92 nmol/L within 2 weeks predicted sustained remission at 5 years. MSeC nadir was the most important prognostic marker following TSS for Cushing’s disease.

## Introduction

Cushing’s disease is a condition of endogenous hypercortisolism caused by an adrenocorticotropin (ACTH)-secreting pituitary adenoma. Prolonged hypercortisolism can lead to significant morbidity and mortality, warranting prompt diagnosis and intervention (Clayton *et al.* 2016). Currently, transsphenoidal surgery (TSS) is the first-line treatment for Cushing’s disease (Nieman *et al.* 2015, Petersenn *et al.* 2015, Bunevicius *et al.* 2019). Determining the adequacy of surgical outcome is dependent on both surgical expertise and biochemical targets used to define remission (Rees *et al.* 2002, Leach *et al.* 2010).

The remission rate after primary surgery from a recent meta-analysis of case series was 80% (95% CI: 77–82%) (Stroud *et al.* 2020), consistent with earlier reviews (Petersenn *et al.* 2015, Bunevicius *et al.* 2019). Lower postoperative morning serum cortisol (MSeC) has been associated with an increased likelihood of remission (Espinosa-De-Los-Monteros *et al.* 2017). However, considerable overlap remains between the postoperative MSeC of patients who experience remission and those who do not (Alwani *et al.* 2010, Sarkar *et al.* 2016). This may be due to centre-dependent differences in postoperative MSeC targets and variations in the timing of postoperative MSeC measurement, which ranges from 7 days to 6 months postoperatively (Stroud *et al.* 2020). The predictive value of other biochemical markers, such as serum ACTH and 24-h urine free cortisol (24-UFC), is less well understood (Costenaro *et al.* 2014, Shirvani *et al.* 2016, Espinosa-De-Los-Monteros *et al.* 2017). There are currently no uniform criteria used to define remission after resection of an ACTH-secreting tumour. According to the 2015 Endocrine Society Guidelines, remission is generally defined as MSeC <138 nmol/L (<5 µg/dL) or 24-UFC <28–56 nmol/day (<10–20 µg/day) within 7 days postoperatively (Nieman *et al.* 2015). The authors of the Endocrine Society Guidelines acknowledge that stricter criteria exist in some centres (eg postoperative serum cortisol <50 nmol/L (<1.8 µg/L) or even <28 nmol/L (<1 µg/dL)), while some clinicians use indirect criteria such as glucocorticoid dependence after surgery (Alahmadi *et al.* 2013, Barbot *et al.* 2013). Optimal timing for assessment of remission is also debated, as it is well described that occasional patients experience delayed hypoadrenalism for a month or more following surgery (Valassi *et al.* 2010). Further complicating assessment is the fact that patients with mild or cyclic Cushing’s disease, or those treated medically prior to surgery with potential for release of corticotroph suppression, may be considered in remission with normal cortisol levels postoperatively (Alexandraki *et al.* 2009, Valassi *et al.* 2018). Developing consensus on the criteria for remission, including timing, is needed as significant management decisions rest on such assessment with some centres performing repeat surgery within a week post initial surgery for patients deemed not to have entered remission (Locatelli *et al.* 2005, Prevedello *et al.* 2008).

If remission is achieved, the risk of recurrence persists for at least 10 years postoperatively (Rollin *et al.* 2007). The recurrence rate of Cushing’s disease in a recent meta-analysis of the literature was 18% (95% CI: 14–22%) during a follow-up of 61.7 ± 31.4 months (Stroud *et al.* 2020). Recurrence was more likely in patients with macroadenomas compared with microadenomas (Dimopoulou *et al.* 2014, Nieman *et al.* 2015, El Asmar *et al.* 2018).

Increased postoperative MSeC has been widely reported as a predictor of recurrence, although no agreed cut-off for MSeC at a consistent time-point postoperatively has been applied to distinguish patients at high vs low risk of recurrence (Khalil *et al.* 2011, Ciric *et al.* 2012, Dimopoulou *et al.* 2014). Many advocate for an undetectable postoperative MSeC as the optimum target (Alexandraki *et al.* 2013, Bansal *et al.* 2015, Stroud *et al.* 2020). However, sustained remission has frequently been reported in patients who remained eucortisolemic during the postoperative period, so the target value remains debatable (Trainer *et al.* 1993, Rees *et al.* 2002, Salenave *et al.* 2004, Testa *et al.* 2007).

Second-line treatments, including revision surgery, may be considered in patients with persistence or recurrence of Cushing’s disease after primary surgery. Compared with primary surgery, remission is less likely after a revision procedure (Benveniste *et al.* 2005, Pouratian *et al.* 2007, Dimopoulou *et al.* 2014, Espinosa-De-Los-Monteros *et al.* 2017). If remission does occur, recurrence rates tend to be higher, and relapses occur sooner than after primary surgery (Dimopoulou *et al.* 2014).

Given the significant consequences of untreated Cushing’s disease, the ongoing evolution of surgical technology and a growing suite of second-line therapies, it is vital for clinicians to be well-informed regarding outcomes of TSS for Cushing’s disease at their centre. The broad objectives of our study were to describe the rates of remission and recurrence after TSS for Cushing’s disease at a tertiary referral centre. It was hoped that identifying predictive factors in our cohort associated with the long-term outcome would assist in planning management and prognostication for individual patients. We were also interested in whether early (<2 weeks) measurement of MSeC was predictive of remission as defined by measurement of MSeC at 6 weeks postoperatively. Finally, the ongoing uncertainty around the importance of achieving an undetectable postoperative MSeC and the clinical consequences of hypocortisolism prompted us to examine whether a less stringent MSeC threshold post surgery would provide confidence in predicting sustained remission.

## Materials and methods

### Study design

A retrospective case series was conducted involving patients who underwent TSS for Cushing’s disease at St Vincent’s Hospital, Sydney (public and private campuses), between 1990 and 2019. Ethics approval was obtained from the SVH Human Research Ethics Committee (LNR/14/SVH/94, LNR/13/SVH/74 and 2019/PID13822).

### Population

Medical records of consecutive adult patients (age ≥ 18 years) undergoing TSS for ACTH producing pituitary adenomas were reviewed. Patients without a definitive diagnosis of Cushing’s disease after surgery, less than 6 months of follow-up or insufficient data to determine postoperative outcome were excluded. Data collected included demographic features, tumour characteristics, prior treatment, pre- and postoperative biochemistry and surgical findings. The goal of the surgery was gross total adenoma resection.

### Preoperative evaluation

Patients were diagnosed with Cushing’s disease if they exhibited clinical manifestations of hypercortisolism and a positive result in at least two of three tests: elevated 24-UFC (>upper limit of normal (ULN)), lack of suppression after low-dose dexamethasone (LDDST; MSeC >50 nmol/L or >1.8 µg/dL) and/or elevated midnight salivary cortisol (>ULN). Cushing’s disease was established by normal or elevated ACTH with additional testing such as high-dose dexamethasone suppression testing (suppression >50%) and inferior petrosal sinus sampling (IPSS) was performed to confirm a central source of ACTH. MSeC, 24-UFC and serum ACTH were measured by chemiluminescence immunoassay (UniCel DxI, Beckman Coulter or Immulite 1000, Siemens Healthcare). Salivary cortisol was measured by electrochemiluminescence immunoassay (ECLIA; Cobas e411, Roche).

A dedicated pituitary MRI scan with gadolinium, or CT scan when MRI was not feasible, was performed for each patient. Tumours were classified as microadenomas (<10 mm) or macroadenomas (≥10 mm). Cavernous sinus invasion was determined by review of preoperative imaging by a neuroradiologist.

### Surgical procedure

Endonasal surgical access was either endoscopic or microscopic, with the exclusive use of an endoscope since 2010. Each operation was a collaborative procedure between a neurosurgeon and an ear, nose and throat (ENT) surgeon. Two neurosurgeons and one ENT surgeon at this centre have exclusively conducted the procedure since 2012.

### Postoperative evaluation

Postoperative biochemistry included MSeC (collected between 08:00 and 10:00 h), as well as serum cortisol after LDDST, serum ACTH and 24-UFC measured within 3 months after surgery. Postoperative nadirs were recorded as the lowest values during the first 6 weeks after surgery, unless stated otherwise. Laboratory reference ranges for MSeC varied, with lower limits between 120 nmol/L (4.4 µg/dL) and 200 nmol/L (7.3 µg/dL). To interpret results consistently, all values <200 nmol/L (<7.3 µg/dL) were considered to represent hypocortisolism. Routine glucocorticoids were not prescribed peri- or postoperatively. Criteria for administering postoperative glucocorticoids were: (1) MSeC <150 nmol/L (<5.4 µg/dL), or (2) symptoms of hypocortisolemia with MSeC <200 nmol/L (<7.3 µg/dL). Glucocorticoids were subsequently weaned until the hypothalamo-pituitary-adrenal axis recovered. For patients on glucocorticoids, MSeC and 24-UFC were assessed at least 24 h after the last dose. MRI scans were conducted within 6 months postoperatively. In most patients, this occurred on postoperative day 1 and 3 months after surgery. Any definitive tumour presence was recorded as a residual.

### Histopathological evaluation

Immunohistochemistry was used to assess tumour expression of ACTH, galanin and other anterior pituitary hormones. Proliferative potential was assessed using the Ki67 index and p53 immunoexpression.

### Complications

Surgical complications were recorded if they occurred within 30 days after surgery. These included cerebrospinal fluid (CSF) leak, meningitis, intracranial haemorrhage (requiring treatment), epistaxis, new visual field disturbance or cranial nerve deficits and death. Medical complications recorded were deep vein thrombosis and pulmonary embolism. Endocrinological complications were recorded as assessed by pituitary hormone testing within 6 weeks after surgery, except in the case of confirmed growth hormone (GH) deficiency where stimulation testing may not have occurred until up to 1 year after surgery. Transient diabetes insipidus (DI) was recorded where treatment was required within 1 week after surgery and there was recovery during follow-up vs new permanent DI, which persisted for the duration of follow-up. The syndrome of inappropriate secretion of antidiuretic hormone (SIADH) was defined by transient hyponatraemia within first 2 weeks after surgery.

### Outcome measures

Initial remission was defined as a nadir MSeC <138 nmol/L (<5 µg/dL) within 6 weeks postoperatively. Persistent disease was diagnosed in cases failing to fulfil these criteria. Sustained remission was defined as the absence of persistent disease or recurrence beyond 6 weeks postoperatively without additional treatment. Recurrence was diagnosed in patients with clinical suspicion of recurrence in combination with biochemical evidence on at least two of three tests, after a documented remission: elevated (>ULN) 24-UFC, lack of serum cortisol suppression following LDDST and/or elevated midnight salivary cortisol.

### Statistical analysis

Analyses were performed using SPSS v25.0 (IBM Corporation). A two-sided *P* value <0.05 was considered significant. Where parametrically distributed, nominal variables were analysed using Fisher’s exact test and continuous variables using Student’s *t* test, reported as mean ± s.d.

Receiver operating characteristic (ROC) curves were constructed and the maximal value of Youden’s index was used to determine optimal cut-off values for postoperative MSeC nadir in predicting sustained remission. A survival analysis was conducted using the Kaplan–Meier estimate.

## Results

### Population characteristics

The study population included 42 patients (age 46.9 ± 13.0 years, 83% female; Table 1). Primary surgery was performed in 30 patients and revision surgery in 12 patients. The preoperative imaging modality was CT in 4 patients (10%) and MRI in 38 (90%). Surgical access was endoscopic in 57% of primary operations and 92% of revision operations (*P* = 0.02). Postoperative follow-up was 54.6 ± 56.0 months. Baseline characteristics of primary and revision groups were similar, except for a higher incidence of cavernous sinus invasion and endoscopic access among revision surgery candidates (42% vs 7%, *P* = 0.01; 92% vs 57%, *P* = 0.04).

**Table 1:** Baseline population characteristics.

Characteristic	All	Primary TSS	Revision TSS	*P* value*a*
Number	42	30	12	
Age at surgery (years)	46.9 ± 13.0	44.8 ± 13.4	52.0 ± 11.0	0.11
Female (%)	83	87	75	0.39
Endoscopic surgical access (%)	67	57	92	0.04*
Postoperative follow-up (months)	54.6 ± 56.0	57.8 ± 57.7	46.6 ± 53.2	0.56
Tumour evident on preoperative MRI (%)	81	83	75	0.67
Lesion size on MRI				
Maximal diameter (mm)	9.3 ± 9.1	8.9 ± 8.1	10.4 ± 11.5	0.64
Microadenoma (<10 mm) (%)	67	67	67	1.00
Tumour invasiveness:				
Cavernous sinus invasion (%)	17	7	42	0.01*
Sphenoid sinus invasion (%)	5	3	8	0.50
Optic chiasm compression (%)	12	10	17	0.61
Preoperative biochemistry:				
MSeC (nmol/L)	579.6 ± 254.7	567.0 ± 287.4	604.9 ± 180.7	0.68
ACTH (pmol/L)	24.0 ± 17.7	24.8 ± 19.3	22.0 ± 13.5	0.67
24-UFC (nmol)	799.0 ± 1021.8	939.3 ± 1199.9	518.4 ± 427.6	0.25

*Statistically significant (*P* < 0.05). ^a^Primary vs revision surgery.

ACTH, adrenocorticotropic hormone; MSeC, morning serum cortisol; TSS, transsphenoidal surgery; 24-UFC, 24-h urine free cortisol.

### Surgical outcomes

Initial remission occurred in 67% of patients overall. The remission rate among patients undergoing primary surgery was 77%, compared with 42% for revision surgery. Recurrence rates were similar for primary and revision surgery patients (17% vs 20%; *P* = 1.00) (Tables 2 and 3). Operations undertaken with endoscopic access were followed by initial remission in 75% of patients, compared with 50% of those performed with microscopy (*P* = 0.17).

**Table 2:** Outcomes of primary transsphenoidal surgery for Cushing’s disease.

Variable	Initial Remission	Persistent Disease	*P* value*a*	Sustained Remission	Recurrence	*P* value*b*
Number (%)	23 (77)	7 (23)		19 (83)	4 (17)	
Age at surgery (years)	42.0 ± 13.6	53.9 ± 7.8	0.04*	42.0 ± 14.2	42.3 ± 12.1	0.97
Female (*n*, %)	21 (91)	5 (71)	0.23	17 (90)	4 (100)	1.00
Length of follow-up (months)	45.6 ± 35.8	97.9 ± 94.5	0.20	35.2 ± 22.7	95.4 ± 47.9	<0.01*
Tumour on preoperative imaging (*n*, %)	20 (87)	5 (71)	0.57	16 (84)	4 (100)	1.00
Tumour visualisation intraoperatively (*n*, %)	23 (100)	6 (86)	0.23	19 (100)	4 (100)	N/A
IPSS lateralisation concordant with tumour location (*n*, %)*c*	3 (100)	0 (0)	0.20	3 (100)	N/A	N/A
Lesion size on MRI:						
Maximal diameter (mm)	7.7 ± 5.4	13.5 ± 14.4	0.38	6.9 ± 5.3	11.7 ± 5.0	0.11
Microadenoma (<10 mm) (*n*, %)	17 (74)	3 (43)	0.18	15 (79)	2 (50)	0.27
Tumour invasiveness:						
Cavernous sinus invasion (*n*, %)	1 (4)	1 (14)	0.42	1 (5)	0 (0)	1.00
Sphenoid sinus invasion (*n*, %)	0 (0)	1 (14)	0.23	0 (0)	0 (0)	N/A
Optic chiasm compression (*n*, %)	2 (9)	1 (14)	1.00	2 (11)	0 (0)	1.00
Preoperative biochemistry:						
MSeC (nmol/L)	519.3 ± 249.5	805.5 ± 384.5	0.07	511.9 ± 246.3	561.0 ± 321.1	0.76
ACTH (pmol/L)	21.0 ± 17.1	38.1 ± 22.4	0.05	20.5 ± 16.8	23.8 ± 22.6	0.78
24-UFC (nmol)	896.6 ± 1266.3	1152.8 ± 899.4	0.71	930.6 ± 1373.7	703.3 ± 269.7	0.49
Postoperative biochemistry:						
MSeC <200 nmol/L within 2 weeks (*n*, %)	23 (100)	2 (25)	<0.01*	19 (100)	4 (100)	N/A
MSeC 2-week nadir (nmol/L)	45.1 ± 47.7	273.3 ± 110.8	<0.01*	42.1 ± 48.4	62.0 ± 45.1	0.54
MSeC 6-week nadir (nmol/L)	26.0 ± 35.5	347.6 ± 220.2	<0.01*	23.8 ± 32.0	36.8 ± 53.8	0.52
ACTH 6-week nadir (pmol/L)	2.4 ± 2.5	6.3 ± 2.6	0.01*	2.2 ± 2.1	3.5 ± 4.9	0.55
24-UFC 6-week nadir (nmol)	27.8 ± 45.0	405.6 ± 522.5	0.11	20.8 ± 46.5	63.0 ± 0.0	0.45
Normal 24-UFC by 6 weeks	23 (100)	3 (60)	0.18	19 (100)	4 (100)	N/A
Tumour histopathology:						
Histological evidence of adenoma (*n*, %)	21 (91)	6 (86)	1.00	17 (90)	4 (100)	1.00
p53 positivity (*n*, %)	19 (78)	7 (100)	1.00	5 (71)	4 (100)	1.00
Ki67 (*n*, %)	2.4 ± 2.4	N/A	N/A	2.7 ± 2.5	1.3 ± 1.7	0.34
Postoperative glucocorticoid requirement (*n*, %)	21 (91)	7 (100)	1.00	18 (95)	3 (75)	0.32

^a^Initial remission vs persistent disease. ^b^Sustained remission vs recurrence. ^c^Data available for four patients. *Statistically significant (*P* < 0.05).

ACTH, adrenocorticotropic hormone; MSeC, morning serum cortisol; N/A, not available (insufficient data); 24-UFC, 24-h urine free cortisol.

**Table 3:** Outcomes of revision transsphenoidal surgery for Cushing’s disease.

Variable	Initial remission	Persistent disease	*P* value^a^	Sustained remission	Recurrence	*P* value^b^
Number (%)	5 (42)	7 (58)		4 (80)	1 (20)	
Age at surgery (years)	45.8 ± 8.5	56.4 ± 11.0	0.10	48.0 ± 8.0	37.0 ± 0.0	0.31
Female (*n*, %)	5 (100)	4 (57)	0.21	4 (100)	1 (100)	N/A
Length of follow-up (months)	19.3 ± 15.5	66.1 ± 62.9	0.14	20.5 ± 17.7	14.6 ± 0.0	0.78
Tumour on preoperative imaging (*n*, %)	3 (60)	6 (86)	0.52	3 (75)	0 (0)	0.40
**Lesion size on MRI**						
Maximal diameter (mm)	3.3 ± 2.0	15.4 ± 13.0	0.05*	4.1 ± 0.9	0.0 ± 0.0	0.02*
Microadenoma (<10 mm) (*n*, %)	5 (100)	3 (43)	0.08	4 (100)	1 (100)	N/A
**Tumour invasiveness**						
Cavernous sinus invasion (*n*, %)	0 (0)	5 (71)	0.03*	0 (0)	0 (0)	N/A
Sphenoid sinus invasion (*n*, %)	0 (0)	1 (14)	1.00	0 (0)	0 (0)	N/A
Optic chiasm compression (*n*, %)	0 (0)	2 (29)	0.47	0 (0)	0 (0)	N/A
**Preoperative biochemistry**						
MSeC (nmol/L)	498.4 ± 124.4	681.0 ± 182.6	0.08	448.5 ± 63.4	698 ± 0.0	0.04*
ACTH (pmol/L)	8.4 ± 3.4	29.8 ± 10.3	<0.01*	7.8 ± 3.9	10.1 ± 0.0	0.67
24-UFC (nmol)	378.2 ± 300.3	618.6 ± 497.0	0.36	261.8 ± 172.6	844.0 ± 0.0	0.06
**Postoperative biochemistry**						
MSeC <200nmol/L within 2 weeks (*n*, %)	5 (100)	2 (29)	0.06	4 (100)	1 (100)	N/A
MSeC 2-week nadir (nmol/L)	81.0 ± 26.9	520.9 ± 323.6	0.01*	85.5 ± 28.8	63.0 ± 0.0	0.53
MSeC 6-week nadir (nmol/L)	70.0 ± 45.2	408.1 ± 305.3	0.03*	71.8 ± 52.0	63.0 ± 0.0	0.89
ACTH 6-week nadir (pmol/L)	4.4 ± 3.0	14.2 ± 10.1	0.10	3.8 ± 3.3	6.1 ± 0.0	0.61
24-UFC 6-week nadir (nmol)	N/A	232.5 ± 221.2	N/A	N/A	N/A	N/A
**Tumour histopathology**						
Histological evidence of adenoma (*n*, %)	5 (100)	7 (100)	N/A	4 (100)	1 (100)	N/A
p53 positivity (*n*, %)	N/A	1 (33)	N/A	N/A	N/A	N/A
Ki67 (*n*, %)	2.6 ± 3.1	6.3 ± 6.5	0.31	3.4 ± 3.3	0.4	0.52
Postoperative glucocorticoid requirement (*n*, %)	4 (80)	2 (29)	0.24	3 (75)	1 (100)	1.00
Residual tumour on 3-month MRI (*n*, %)	0 (0)	2 (40)	0.44	0 (0)	0 (0)	N/A

^a^Initial Remission vs persistent disease. ^b^Sustained remission vs recurrence. *Statistically significant (*P* < 0.05).

ACTH, adrenocorticotropic hormone; MSeC, morning serum cortisol; N/A, not applicable (insufficient data); 24-UFC, 24-h urine free cortisol.

#### Primary surgery: initial remission vs persistent disease

Initial remission occurred in 77% of primary surgery candidates. Initial remission was significantly more likely in patients with MSeC < 200 nmol/L (<7.3 µg/dL) within 2 weeks postoperatively (100% vs 25%, *P* < 0.01), lower postoperative MSeC nadir (26.0 ± 35.5 nmol/L vs 347.6 ± 220.2 nmol/L, *P* < 0.01) and ACTH (2.4 ± 2.5 pmol/L vs 6.3 ± 2.6 pmol/L, *P* = 0.01), and older age at surgery (53.9 ± 7.8 years vs 42.0 ± 13.6 years, *P* = 0.04) (Table 2). There was no statistically significant relationship between initial remission and tumour size, tumour visibility or cavernous sinus invasion on preoperative imaging, gender, age at surgery, pre-operative biochemistry, histological evidence of adenoma, pathological markers (Ki67, p53) or requirement for glucocorticoid replacement. In patients with initial remission, there was a trend towards lower preoperative MSeC (519.3 ± 249.5 vs 805.5 ± 384.5, *P* = 0.07) and ACTH (21.0 ± 17.1 vs 38.1 ± 22.4, *P* = 0.05). No patient undergoing primary surgery had residual tumour visible on the postoperative 3-month MRI, regardless of remission status.

IPSS showed lateralisation of ACTH secretion in response to corticotropin-releasing hormone in four patients. Intraoperative tumour location was concordant with IPSS results in three of these, all of whom experienced remission. The patient in whom IPSS did not correspond to tumour location had a persistent disease (Table 2). In patients with negative preoperative imaging, the concordance between IPSS and intraoperative tumour location was lower. Among the four patients who underwent primary surgery with negative preoperative imaging, there was lateralisation of ACTH response in only two patients, which corresponded with intraoperative tumour location in one case.

**Figure 1:**
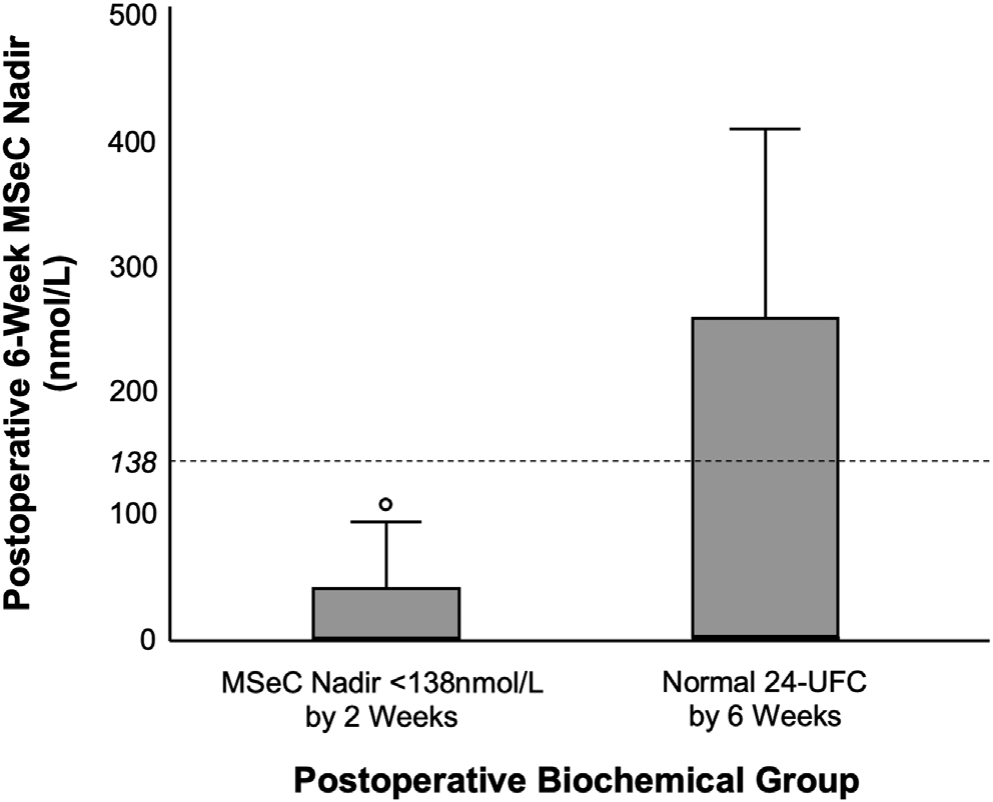
Patients in remission after primary transsphenoidal surgery for Cushing’s disease. Comparison of MSeC nadir <138 nmol/L by 2 weeks postoperatively and achievement of normal 24-UFC by 6 weeks vs 6-week MSeC nadir. MSeC, morning serum cortisol; 24-UFC, 24-h urine free cortisol.

All patients with nadir MSeC <138 nmol/L (<5 µg/dL) within 6 weeks postoperatively also demonstrated a normal 24-UFC at 6 weeks. However, at 6 weeks postoperatively, 33% (3/9) of patients with normal 24-UFC still had a nadir MSeC >138 nmol/L (>5 µg/dL; Fig. 1). One of these had a 6-week nadir MSeC of 408 nmol/L (14.8 µg/dL) but experienced clinical resolution of Cushing’s disease. He subsequently experienced recurrence 9 years and 3 months after primary surgery. Among the six patients with both a normal 24-UFC and MSeC <138 nmol/L (<5 µg/dL) at 6 weeks, only one experienced a recurrence. Hence, patients with both normal 24-UFC and an MSeC nadir <138 nmol/L (<5 µg/dL) by 6 weeks were less likely to experience recurrence than those with normal 24-UFC but an MSeC nadir >138 nmol/L (>5 µg/dL).

Among those who met the criteria for initial remission of MSeC <138 nmol/L (<5 µg/dL) by 6 weeks postoperatively, all but one had reached this level within 2 weeks. This patient subsequently experienced an MSeC level of 77 nmol/L (0.3 µg/dL) on day 19. Hence, a 2-week MSeC nadir <138 nmol/L (<5 µg/dL) had a sensitivity of 95% and specificity of 100% for remission at 6 weeks after surgery. Among patients with initial remission, there was a statistically significant reduction in nadir MSeC values between 2 and 6 weeks after surgery (mean difference: −15.2 ± 31.1 nmol/L, *P* = 0.04).

#### Primary surgery: sustained remission vs recurrence

**Figure 2:**
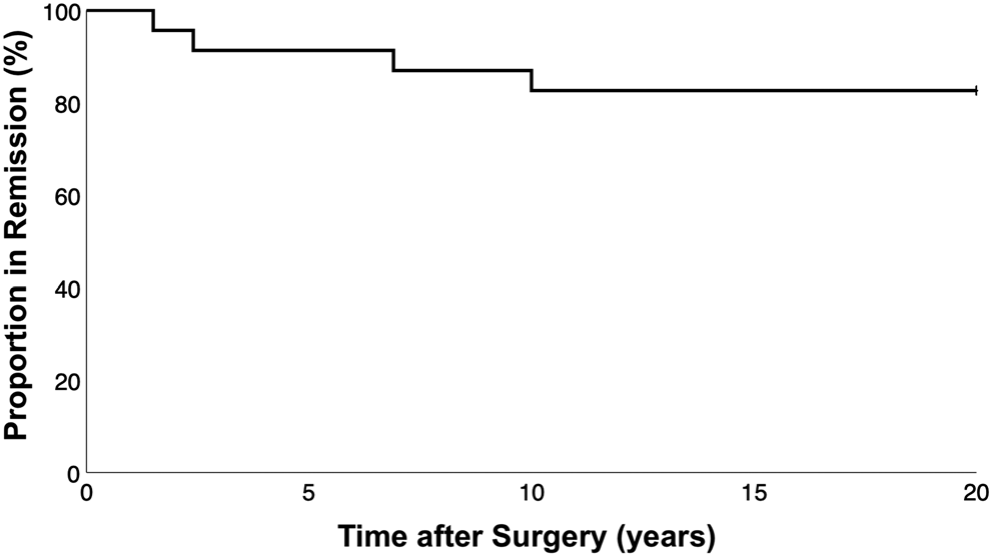
Kaplan–Meier survival analysis of the proportion of patients remaining in remission after primary transsphenoidal surgery for Cushing’s disease.

Of 23 patients experiencing initial remission, 17% had recurrence (Fig. 2). There was a significantly longer follow-up period among those with documented recurrence vs those in sustained remission, due to a statistical outlier with 162.2 months of postoperative follow-up (95.4 ± 47.9 months vs 35.2 ± 22.7 months, *P* < 0.01; Table 2). Mean time to recurrence was 5.2 ± 4.0 years.

**Figure 3:**
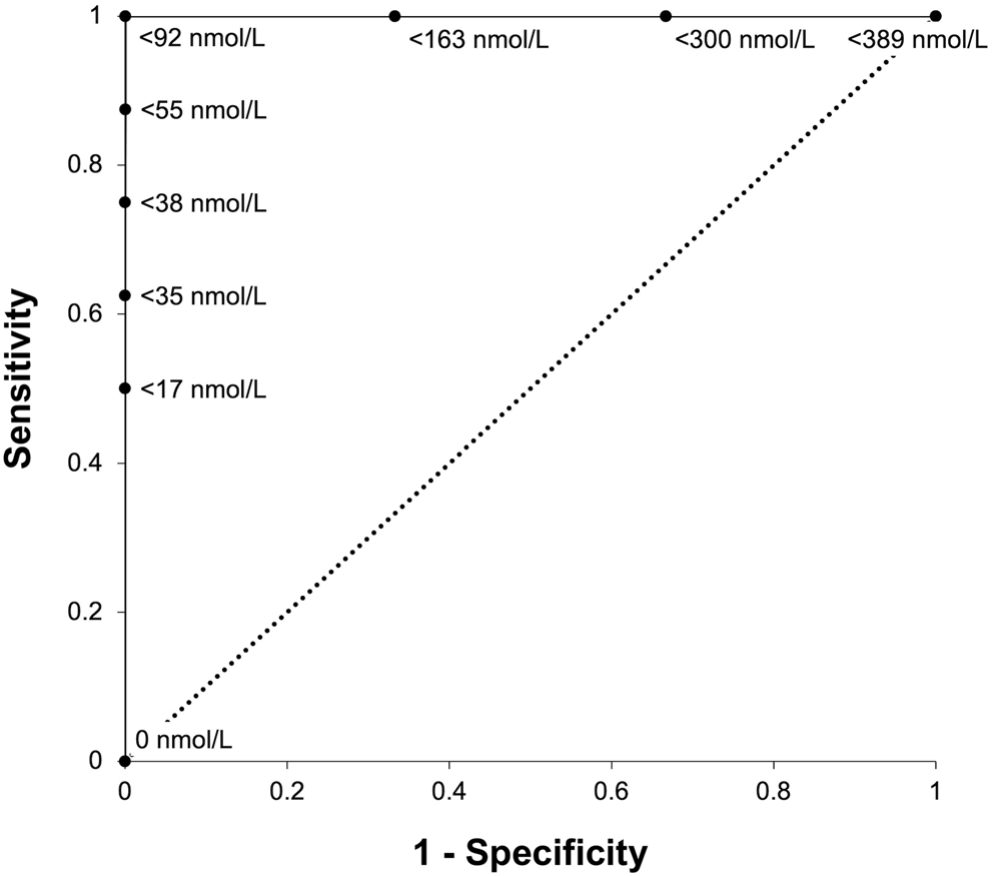
Receiver operating characteristic curve showing the statistical performance of different morning serum cortisol cut-offs at 2 weeks postoperatively for predicting sustained remission at 5 years.

Sustained remission for at least 5 years postoperatively was predicted by lower MSeC nadir, regardless of whether this was reached within 2 weeks or 6 weeks after surgery. On ROC analysis, an MSeC nadir <92 nmol/L (<3.3 µg/dL) at either 2 or 6 weeks postoperatively had 100% sensitivity and specificity for predicting sustained remission at 5 years. The performance of different 2-week MSeC nadir cut-offs for predicting sustained remission at 5 years is shown in Fig. 3. Compared to patients who experienced recurrence, those in sustained remission at 5 years after surgery had significantly lower MSeC nadirs at 2 weeks (22.4 ± 26.4 nmol/L vs 237.7 ± 138.9 nmol/L, *P* < 0.01) and at 6 weeks (17.5 ± 26.5 nmol/L vs 329.0 ± 274.6 nmol/L, *P* < 0.01). Three patients had a postoperative MSeC nadir between 92 nmol/L (3.3 µg/dL, the optimum cut-off for predicting sustained remission) and 138 nmol/L (5 µg/dL). Of these, one patient (with a postoperative MSeC nadir of 114 nmol/L (4.1 µg/dL)) experienced recurrence after 18 months.

Sustained remission was not predicted by age at surgery, gender, visible tumour on preoperative MRI, tumour size, sphenoid sinus invasion, preoperative biochemistry, postoperative ACTH or 24-UFC, histological evidence of tumour, pathological markers (Ki67, p53) or postoperative glucocorticoid replacement.

#### Revision surgery

Twelve patients underwent revision surgery. Seven had undergone primary surgery at the reporting centre and five had their primary operations elsewhere. Initial remission occurred in 5 out of 12 cases (42%; Table 3). Compared to those with persistent disease, patients with initial remission had smaller maximal tumour diameter (3.3 ± 2.0 mm vs 15.4 ± 13.0 mm, *P* = 0.05), lower incidence of cavernous sinus invasion (0% vs 71%, *P* = 0.03), lower preoperative ACTH (8.4 ± 3.4 pmol/L vs 29.8 ± 10.3 pmol/L, *P* < 0.01) and lower postoperative MSeC nadir (70.0 ± 45.2 nmol/L vs 408.1 ± 305.3 nmol/L, *P* = 0.03). One out of five patients with initial remission experienced recurrence. This occurred 1.1 years after surgery and has been adequately controlled with cabergoline for at least 12 months since. The patient who experienced recurrence presented with higher preoperative MSeC compared with the four patients who remained in remission (698 nmol/L vs 443.2 ± 56.2 nmol/L, *P* = 0.04). She did not have a visible tumour on preoperative imaging, although the presence of an ACTH-secreting adenoma was confirmed on histopathology.

### Complications

There were no deaths related to surgery. The most frequent endocrinological complication was transient DI, which occurred in 23% of patients undergoing primary surgery and 25% undergoing revision surgery (Table 4). Surgical complications following primary surgery occurred in 3% of patients, consisting exclusively of CSF leak in one patient. Intracranial haemorrhage occurred in two patients undergoing revision surgery.

**Table 4:** Complications related to transsphenoidal surgery for Cushing’s disease

Complication	Prevalence		
	Primary surgery	Revision surgery	*P* value
Number undergoing surgery	30	12	
Any endocrine complications (*n*, %)	10 (33)	5 (42)	0.73
Transient DI (*n*, %)	7 (23)	3 (25)	1.00
Permanent DI (*n*, %)	2 (7)	1 (8)	1.00
SIADH (*n*, %)	2 (7)	1 (8)	1.00
New hypothyroidism (*n*, %)	0 (0)	1 (8)	0.47
New GnT deficiency (*n*, %)	0 (0)	0 (0)	N/A
New GH deficiency (*n*, %)	0 (0)	0 (0)	N/A
Any surgical complications (*n*, %)	1 (3)	2 (17)	0.19
CSF leak (*n*, %)	1 (3)	0 (0)	1.00
Haemorrhage (*n*, %)	0 (0)	2 (17)	0.08
New visual field defects (*n*, %)	0 (0)	0 (0)	N/A
New cranial nerve deficit (*n*, %)	0 (0)	0 (0)	N/A
Epistaxis (*n*, %)	0 (0)	0 (0)	N/A
Meningitis (*n*, %)	0 (0)	0 (0)	N/A
Death (*n*, %)	0 (0)	0 (0)	N/A

CSF, cerebrospinal fluid; DI, diabetes insipidus; GH, growth hormone; GnT, gonadotropin; SIADH, syndrome of inappropriate antidiuretic hormone secretion.

## Discussion

This series described the outcomes of transsphenoidal surgery for Cushing’s disease at a tertiary referral centre. The remission rate after primary surgery (77%) was consistent with recent studies using a similar MSeC cut-off to define remission (Ammini *et al.* 2011, Kuo *et al.* 2015, Bansal *et al.* 2017, Espinosa-De-Los-Monteros *et al.* 2017), as was the remission rate after revision surgery (42%) (Espinosa-De-Los-Monteros *et al.* 2017, Johnston *et al.* 2017, Mayberg *et al.* 2018). Hypotheses for lower remission rates after revision surgery include alteration of surgical landmarks during the primary procedure, the development of scar tissue making complete resection more challenging and more aggressive biology of tumours that persist or recur after initial surgery (Pouratian *et al.* 2007, Valderrabano *et al.* 2014). In this series, the higher incidence of tumour invasion into adjacent structures among patients undergoing revision surgery supports the latter hypothesis.

The recurrence rate after primary surgery (17%) was consistent with that reported in several recent series (Cebula *et al.* 2017, Johnston *et al.* 2017, Feng *et al.* 2018). For revision surgery, the recurrence rate (20%) was lower than that reported in several recent studies with a comparable sample size (Valderrabano *et al.* 2014, Espinosa-De-Los-Monteros *et al.* 2017, Johnston *et al.* 2017). This may be due to differences in follow-up duration. However, it is difficult to be certain due to variability in follow-up data reported. One study did not report the follow-up duration for all of their revision surgery patients (Johnston *et al.* 2017). The second had a longer follow-up duration, a median of 2.5 years (IQR 1.8–5.1) (Espinosa-De-Los-Monteros *et al.* 2017). The third study had a median follow-up of only 13 months (range, 2–18 months) (Valderrabano *et al.* 2014). The recurrence rate of 20% was also lower than the recurrence rate of 28% reported after revision surgery in a recent meta-analysis (Stroud *et al.* 2020).

The results of this study reaffirm the utility of postoperative MSeC nadir for predicting remission in patients undergoing primary surgery for Cushing’s disease. MSeC <200 nmol/L (<7.3 µg/dL) within 2 weeks after surgery was strongly predictive of subsequent remission, with a sensitivity of 100%, specificity of 75% and positive predictive value (PPV) of 95%. Patients in this study had serum cortisol measurements reported by a variety of assays and instruments of varying sensitivity. However, all measurements were performed using chemiluminescence assays and the results are supported by several prior studies. The same cut-off (<200 nmol/L or <7.3 µg/dL) within 2 weeks postoperatively yielded a PPV for remission of 97% in another case series (Alwani *et al.* 2010). In the present series, the more rigorous MSeC cut-off of <92 nmol/L (<3.3 µg/dL), also obtained within 2 weeks postoperatively, accurately identified patients remaining in long-term remission with a PPV of 100% at 5 years after surgery (sensitivity and specificity of 100%). A similar cut-off of <97 nmol/L (<3.5 µg/dL) within 48 h of surgery had a specificity and PPV of 100% for remission in another study (Costenaro *et al.* 2014). These results suggest that postoperative MSeC monitoring can be confidently used to establish remission within 2 weeks after surgery. Recent guidelines support the earlier determination of remission, referring to a threshold of MSeC <138 nmol/L (<5 µg/dL) or 24-UFC <56 nmol (<2 µg/dL) achieved within 7 days of selective adenomectomy (Nieman *et al.* 2015).

Some prior studies have used a relatively low threshold of <50 nmol/L (<1.8 µg/dL) or undetectable MSeC postoperatively as an indicator of remission (Alwani *et al.* 2010, Ammini *et al.* 2011, Hassan-Smith *et al.* 2012). Whilst a postoperative MSeC nadir <50 nmol/L (<1.8 µg/dL) has been found to be strongly indicative of sustained remission (Alwani *et al.* 2010), patients may become clinically unwell prior to reaching this level, and it is not clear whether this is necessary to provide clinicians with confidence about long-term remission. We found a higher cut-off of <92 nmol/L (<3.3 µg/dL) provided better sensitivity (100% vs 75%) and equal specificity (100%) compared to <50 nmol/L (<1.8 µg/dL) for identifying patients in sustained remission 5 years after surgery. Three patients who underwent primary surgery had a postoperative MSeC nadir between 92 nmol/L (3.3 µg/dL, the optimum cut-off identified for predicting sustained remission) and 138 nmol/L (5 µg/dL, the cut-off generally proposed per guidelines to define remission (Nieman *et al.* 2015)). Of these, one patient (with a postoperative MSeC nadir of 114 nmol/L or 4.1 µg/dL) experienced a recurrence after 18 months. Hence, we propose that the stricter threshold of <92 nmol/L (<3.3 µg/dL) can be confidently used to predict sustained remission while not necessitating the recording of undetectable MSeC levels postoperatively.

Ancillary tests such as 24-UFC or dexamethasone suppression testing may be useful in confirming remission. Lower postoperative 24-UFC nadir has previously been identified as a predictor of initial remission (Kim *et al.* 2012, Alexandraki *et al.* 2013, Shirvani *et al.* 2016). Nevertheless, 24-UFC may be less valuable as a predictor of long-term outcomes. We found that patients with normal 24-UFC but nadir MSeC >138 nmol/L (>5 µg/dL) at 6 weeks postoperatively had a higher recurrence rate than those with both a normal 24-UFC and an MSeC nadir <138 nmol/L (<5 µg/dL; one in three experienced recurrence, compared to one in six). This supports the importance of a nadir MSeC <138 nmol/L, regardless of normal 24-UFC, for outcome prognostication. However, these results should be interpreted with caution due to the small sample size.

The predictive value of postoperative ACTH has been less extensively studied than serum cortisol. Some previous studies have found significant associations or trends towards an increased likelihood of remission with lower postoperative ACTH (Kim *et al.* 2012, Kuo *et al.* 2017) while others have found no association (Shirvani *et al.* 2016). This study found that in the 6 weeks after primary surgery, nadir ACTH levels were significantly lower in patients with initial remission compared to persistent disease (2.4 ± 2.5 pmol/L vs 6.3 ± 2.6 pmol/L, *P* = 0.01). A similar trend occurred after revision surgery, with patients in initial remission having lower 6-week ACTH nadirs than those with persistence (4.4 ± 3.0 vs 14.2 ± 10.1, *P* = 0.10).

With regards to potential histopathological markers of aggression, this study did not find an association between elevated Ki67 index or p53 immunoreactivity and increased rates of persistence or recurrence. These markers were inconsistently analysed in this series, particularly for patients undergoing surgery prior to 2010. The correlation of these features with tumour behaviour in the literature remains controversial (Di Ieva *et al.* 2014, Syro *et al.* 2015). In a series of 59 patients with Cushing’s disease, increased Ki67 immunoreactivity was associated with a non-significant trend towards lower remission rates, however, this association was lost when tumour volume was taken into account (Witek *et al.* 2016). In a larger series of 82 patients, p53 and Ki67 expression were not significantly associated with initial remission rates (Keskin *et al.* 2017). Other studies have, however, found increased Ki67 expression to be significantly associated with increased risk of recurrence or local invasion, although optimal predictive scores range from as low as 1% through to 3% (Thapar *et al.* 1996, Gejman *et al.* 2008, Righi *et al.* 2012, Trouillas *et al.* 2013, Keskin *et al.* 2017). P53 positivity has not been found to be significantly different between cases with and without recurrence in recent publications, despite large case numbers (Righi *et al.* 2012, Trouillas *et al.* 2013).

Tumour size has previously been identified as a predictor of remission, with higher remission rates observed in microadenomas (Wagenmakers *et al.* 2013, Shirvani *et al.* 2016). This study did not find a statistically significant difference in outcomes between patients with micro- and macroadenomas, although smaller tumour diameter was associated with a higher rate of initial remission after revision surgery (3.3 ± 2.0 mm vs 15.4 ± 13.0 mm, *P* = 0.05). This may reflect the arbitrary distinction between micro- and macroadenomas, as smaller macroadenomas may be just as amenable to successful surgical resection as microadenomas.

The identification of an adenoma on preoperative imaging did not significantly affect outcomes in this series. IPSS was used to investigate adenoma lateralisation in six patients undergoing primary surgery. There was a high rate of IPSS concordance with intraoperative tumour location among patients in remission, regardless of adenoma identification on preoperative imaging (three out of three; Table 2). Among four patients with negative preoperative imaging, there was lateralisation of ACTH response in two patients. A tumour was visualised intraoperatively in both cases, and IPSS lateralisation was concordant with tumour location in one case. Therefore, the utility of IPSS for tumour lateralisation in patients with negative imaging was limited. Nevertheless, IPSS remains an important tool for the confirmation of a central ACTH source.

The presence of cavernous sinus invasion was greater in patients who experienced persistent disease after both primary and revision surgery, although this association only reached statistical significance for revision surgery. The blunting of this difference in the group undergoing primary surgery may have been due to a smaller proportion of patients with cavernous sinus invasion compared to the group undergoing revision surgery, and the majority of patients with cavernous sinus invasion who experienced remission having undergone surgery with endoscopic access (67%). Overall, higher rates of remission were found in those undergoing endoscopic surgery (*n*  = 34) compared with microscopic surgery (*n*  = 8), although statistical significance may have been limited by patient numbers. The panoramic view provided by the endoscope has been shown to improve the rate of gross total resection of pituitary adenomas, but whether this translates into superior long-term outcomes over microscopy remains controversial (D’Haens *et al.* 2009, Alahmadi *et al.* 2013, Gao *et al.* 2014). The effect of a change in surgeons over the years from 1990 to 2019 cannot be excluded, although approximately half of the study population (23 patients) were operated on by the same neurosurgeon and ENT surgeon during the last 8 years of this study’s recruitment period (2012–2019, 2.9 patients per year).

The identification of an adenoma on histopathology has been associated with higher rates of remission in many previous studies (Invitti *et al.* 1999, Hassan-Smith *et al.* 2012, Wagenmakers *et al.* 2013). Whilst prognostically significant, a lack of pathological confirmation of tumour resection is not uncommon, with one large series reporting an incidence of 22.7% (*n* = 111, of 490 patients with Cushing’s disease) (Pouratian *et al.* 2007). In this study, patients may have experienced remission despite negative histopathology due to a high rate of intraoperative tumour visualisation (98% of operations overall, and 80% of those for which histopathology was negative). The absence of tumour tissue on histopathology may have been due to surgical exploration causing necrosis and vascular damage to the adenoma, loss in suction or abnormal tissue being overlooked in the resected specimen (Pouratian *et al.* 2007). In the four patients with negative histopathology, all were diagnosed with pituitary-dependent elevated ACTH prior to surgery: two were found to have macroadenomas on MRI (14 and 17 mm in diameter) in the context of hypercortisolism, and two demonstrated a central to peripheral gradient on IPSS.

For patients who underwent revision surgery, a lower preoperative ACTH was associated with increased likelihood of initial remission, and lower preoperative MSeC was associated with sustained remission. These results have been demonstrated in several prior case series of primary TSS for both ACTH (Rees *et al.* 2002, Espinosa-De-Los-Monteros *et al.* 2017) and MSeC (Kim *et al.* 2012, Shirvani *et al.* 2016). To the authors’ knowledge, this is the first series demonstrating significant predictive value for preoperative ACTH and MSeC in revision TSS. The association between preoperative biochemistry and patient outcomes may reflect more aggressive tumour biology in patients presenting with more severe hypercortisolism, and it is possible in this series that this was most evident in those undergoing revision rather than primary surgery. The lower preoperative MSeC and ACTH values, combined with smaller tumours, in those who experienced a favourable outcome suggests that such patients may be good candidates to guide towards successful revision TSS.

Endocrinological complications of transsphenoidal surgery for Cushing’s disease are frequently under-reported in published studies. In a recent meta-analysis, the rate of surgery-related hypopituitarism was higher for Cushing’s disease than for other functioning pituitary adenomas, however, only 54% of studies reported data about this complication (Roelfsema *et al.* 2012). Greater transparency regarding complications related to surgery is integral to the informed selection of optimal treatments. Hence, this series contributes important information regarding an aspect of treatment for Cushing’s disease which is often overlooked. Endocrinological deficiencies were found to be the most common complication after both primary and revision surgery, predominantly consisting of transient DI (incidence of 23 and 25%, respectively). Intracranial haemorrhage was not a common surgical complication. Both patients who experienced haemorrhage in this series were undergoing revision operations and had invasion into the cavernous sinus, making resection more challenging.

## Conclusions

This study demonstrates high remission rates following TSS for patients with Cushing’s disease in a tertiary referral centre. It has extended previous findings supporting the long-term predictive value of postoperative MSeC nadir in patients undergoing primary surgery. An MSeC nadir of <92 nmol/L (<3.3 µg/dL), reached within 2 weeks of surgery, is proposed to be used with confidence in documenting remission from Cushing’s disease up to 5 years postoperatively. The finding of higher rates of recurrence among macroadenomas along with a lower degree of preoperative hypercortisolism among patients undergoing revision surgery who experience remission may indicate adverse aspects of tumour biology that affect patient outcomes. These findings provide guidance for clinicians regarding the best approach to the postoperative assessment of patients undergoing TSS for Cushing’s disease.

## Declaration of interest

Richard J Harvey is consultant with Medtronic, Stryker, Novartis, Meda, and NeilMed pharmaceuticals. Research grant funding received from Glaxo-Smith-Kline and Stallergenes. He has been on the speakers’ bureau for Glaxo-Smith-Kline, Meda Pharmaceuticals and Seqirus. Ann McCormack has received speaker honorarium for IPSEN, Pfizer and Novartis. Benjamin P Jonker has received speaker fees from Integra LifeSciences Corporation. All other authors have no financial disclosures or conflicts of interest.

## Funding

This work did not receive any specific grant from any funding agency in the public, commercial or not-for-profit sector.

## Ethics approval

All procedures performed in studies involving human participants were in accordance with the ethical standards of the institutional and/or national research committee and with the 1964 Helsinki declaration and its later amendments or comparable ethical standards. Informed consent was obtained from all individual participants included in the study. Ethics approval was granted by the St Vincent’s Hospital Human Research Ethics Committee (reference no. LNR/14/SVH/94 and LNR/13/SVH/74).

## Author contribution statement

A S: Manuscript drafting, data acquisition and analysis. P D: Manuscript drafting, data acquisition and analysis. R J H: Study conception and design, data interpretation, manuscript revision. R A: Data interpretation, manuscript revision. M W: Study conception, data analysis, manuscript revision. B P J: Study conception, data analysis, manuscript revision. J G: Data interpretation, manuscript revision. A M: Study conception and design, data interpretation, manuscript revision.
